# American Academy of Dental Science

**Published:** 1893-02

**Authors:** 

**Affiliations:** American Academy of Dental Science


### AMERICAN ACADEMY OF DENTAL SCIENCE.

The regular monthly meeting of the American Academy of Dental Science was held in the Boston Medical Library Association rooms, on October 5, 1892, at 7.30 P.M., President Brackett in the chair.

Brief discussions were held upon the following subjects: "Banding Fractured Roots," and "Various Methods of Root-Filling."

### DISCUSSION.

*President Brackett.*—In the absence of other suggestions, it appears in order to begin the discussion of "Banding Fractured Roots."

*Dr. Banfield.*—A patient presented herself to me a few days ago with an upper central which was loose, sore to touch, and had a discharge of pus from its socket. Upon examination and inquiry I found that the root was supporting a banded crown. It had become fractured some years ago, and had been banded in the hope of maintaining it as a useful member.

Considering it useless to attempt the treatment of a root in such a condition, I extracted it, and, to my surprise, found that the process of banding, instead of bringing the fractured parts more closely together, had forced the apex of the fracture against the alveolus. I will pass the specimen around, and you will see how the apex of the slivered portion has been carried out in forcing on the band.

The history of this tooth is this: At first a crown with a wooden pivot was adjusted, and after being worn for some years the end of the root had become weakened, until it finally fractured; then the root was banded, with the result which you see. I present this specimen to you to show how easily we may be deceived in similar operations.

In the treament of fractured roots, I first tie a ligature around the root, bringing the parts closely together; then make an application of compound tincture of benzoin to the inflamed gums, and wait twenty-four to forty-eight hours. As soon as the soreness has sufficiently subsided, I drill a small hole through the fractured portions of the root near the gum and insert a gold screw. If there is a filling, it is replaced with Weston's cement, and care is taken that the opposing tooth does not strike the fracture in the act of mastication. The ligature can then be removed. After the parts are held together awhile by the screw and cement, the inflammation ought to entirely subside. The crown or remaining portion can then be removed or so shaped as to receive a band or cap. This method applies to fractures extending some distance above the gum. If, however, the fracture does not extend too far above the gum, I remove it, and, in fitting the band, cut it so as to completely enclose the space made by the removal of the fractured piece. I recall the case of a young man receiving a blow which fractured a central incisor; the fracture extended fully one-quarter of an inch above the gum on the palatal surface, and this space was completely covered with a portion of the gold band. This operation was performed some ten years ago, and to-day the parts are in a good and healthy condition, thus showing that the gum-tissue will in many cases admit of the band when properly adjusted.

*Dr. Allen.*—While listening to the remarks of Dr. Banfield, and especially in examining the specimen he presents, I could not help being convinced of the fact that it is almost impossible to tell when we have really succeeded in banding a root after the method described, and that a post-mortem examination is the only means we have of determining the result.

I remember a similar case,—that of a lady who, for a number of years, had worn an ordinary crown tooth with a metal post on an upper central incisor. An incautious use of the tooth had split the root, causing inflammation of the socket and loosening of the crown. My treatment consisted in cleansing the parts and banding the fractured portions of the roots firmly together, according to the method just described; after which I reset the crown with oxyphosphate cement, and while the condition, generally, was greatly improved, and has so remained, I do not feel certain that the fractured parts are in correct apposition.

Another case, though somewhat different, was that of a left superior first molar, the fracture extending through the crown, separating the buccal roots from the palatal. As usual in such cases, there was much inflammation, and pus was exuding from between the fractured surfaces. The treatment here consisted in tying a silk ligature around the crown, to hold the parts together, then drilling through the crown cavity, which extended to the pulp-chamber, and opening the canals as well as I could. The canals were found to have been previously filled with a solution of gutta-percha. I then enlarged the canal in the palatal root, and also the canal in the buccal root, and, taking a piece of irido-platinum wire, formed a little staple, which was fitted into the two roots, and which held them tightly together. This staple being cemented into place, the entire crown cavity was filled with oxyphosphate cement, after which I put a gold crown on the tooth. It is now three years since that operation was performed, and the tooth is still doing excellent service.

In cases where roots are slightly fractured one-eighth inch or so beyond the margin of the gum, I remove the broken piece, and make a gold band with a broad flange, covering the edge of the root from which the other piece has been removed. I have also succeeded admirably, in cases where the root has decayed to a high point on one side, by widening the band at that point and then capping the band in the ordinary way.

*Dr. Andrews.*—I remember a case of fractured root which has something of interest in it. Before I went into my present office, nine years ago, a young man had an ordinary pivot tooth put on, which was held in with either cement or gutta-percha, I don't remember which. Soon after, he fractured the root. A band was made of platinum and the pivot tooth again inserted. The platinum not only went around the root, but also part way over the end of it. That held until nearly a year ago,—all of nine years, I should say. For some reason the tooth became loosened, and while I was away my associate reset it again with red gutta-percha. That held for about two or three months, then came out again, and upon looking at the band closely I came to the conclusion that pressure had enlarged it, as blood oozed from between the fractured parts when moved. I took off the platinum casing or band, and with wire brought the parts closely together.

The fracture seemed to be from the front, running nearly down to the apex of the root. I then made another band, this time of gold, with a lip wider in front, to go upon the root, fitted it in as accurately as I could and burnished into place. Before putting it on, however, I dried out the root thoroughly, using gum sandarac, and put the tooth on with oxyphosphate. The tooth, apparently, is working very well.

*Dr. Stevens.*—In examining the tooth that Dr. Banfield passed around, it looks to me as if the fracture may have been caused by the insertion of the pivot rather than by the drawing in of the band.

*Dr. Banfield.*—I can't say in regard to that. It may have been decayed to such an extent that it could not be brought together.

*Dr. Grant.*—It seems to me that the tooth presents about the same features that any fractured root would, and I never believed it possible for any one to bring the two parts of a fractured incisor together and make a joint. The very thing which shows in this specimen is the very thing I should expect,—by pushing on an inclined plane the point of the fracture is thrown off. I have always in such cases cut the fractured part off at once. If it seemed to be short enough, and if the upper edge of the fracture could be reached by a lip on the band, I run the lip up there, and I have in one or two instances put a band on the end of the root and used it as a matrix, putting an amalgam filling in it, and then taking the band off. I am not a believer in bands in the front part of the mouth. I have yet to see the banded incisor root that did not become a defacement, particularly in people who have passed middle age. You may put them on young people and, if circumstances are favorable, and there is no tendency to recession of the gums, they do not show, but if the patient is over forty they are apt to show within a couple of years. I don't believe it is possible to repair the fracture of an incisor by trying to bring the pieces into apposition.

*Dr. Banfield.*—I would like to ask Dr. Grant, in a case of this kind, but with a clean fracture, how he would treat it except by putting on a band?

*Dr. Grant.*—I would take it out if I could.

*Dr. Banfield.*—Then you would have to extract the root.

*Dr. Grant.*—That's just what happened there. All things which are possible are not desirable. If such a root as has been described is banded or repaired, as the dentist thought he repaired it, and the patient is tortured three or four years, I don't call that good dentistry. My idea of good dentistry is to do the very best that can be done, and not attempt impossible things.

*Dr. Banfield.*—To illustrate, we have an abscessed tooth, and by treating that tooth we can make it useful for from five to ten years. Now, will you extract a tooth because it may last only five years? The same principle holds in the case of the abscessed tooth and the fractured root.

*Dr. Grant.*—I don't agree with you. I think the chances of success are quite different. If you treat an abscessed tooth, you stand a fair chance of being successful, but in treating a fractured root you are almost sure to fail.

*Dr. Banfield.*—But the tooth which has been shown lasted four years. Here was this fracture which extended more than half-way up the root. Now, it seems to me that a band around a root of this kind, put on with a fair degree of skill, ought to hold the parts together securely and make a useful member. Suppose once in a while we do have a failure, it seems to me the successful operations will well repay us.

*Dr. Williams.*—I have a case of a left central incisor tooth which has been split and banded, or "hooped," as I call it, and it has been doing good service for about ten years.

In regard to what is "good dentistry," my idea is that we must know not only what to do, but how to do it. If you think it is necessary to band a fractured root with a wide, heavy, thick hoop, or section of a barrel you might call it, you make a mistake. You often get sufficient hooping with a fine wire. It keeps the parts of the root together sufficiently well and is all that is needed. In fact, to put on a heavy gold hoop to keep fractured parts together seems to me about as practical as taking a windlass to wind a watch. If the split is long, I would invariably hoop it; if a short one, rarely.

*Dr. Banfield.*—It would be interesting to hear from Dr. Williams how he adjusts this wire and secures it in place.

*Dr. Williams.*—I first wind a fine wire around the tooth, and then adjust gold wire slightly tight, and, after soldering the ends together, spring it over just under the edge of the gum, so that the gum covers the wire. The root is of a sufficiently even size to hold the wire where it is, and the band stays in place by its own tension. The wire used is a non-elastic wire. When in place, there is not tension enough to strain it, and there is no chance of crowding the upper end of the tooth out, as was done in the case presented here to-night.

*President Brackett.*—If no one wishes to add to the very practical discussion that we have had on this subject, we will proceed to the other topic,—"Various Methods of Root-Filling."

*Dr. Allen.*—Root-filling is a matter that I have given especial attention to ever since I began dentistry. I think I have tried nearly all the methods, but, in the main, have adhered to one, and that is the use of oxychloride of zinc. I have not used it invariably, but consider it the best root-filling, and especially in cases of putrescent root-canals. I don't think I have had trouble in more than two per cent. of the cases that I have thus treated, and where I have had trouble I attributed it more to a faulty operation than to the method employed. To overcome the difficulty of sealing the apical foramen, I employ this method. Assuming that the pulp-canal has been thoroughly cleansed, and ventilated for a sufficient length of time to get rid of the products of decomposition, I dry it out with hot air. This is accomplished by taking an ordinary chip-blower and drawing the air through the alcohol- or gas-flame, and inserting the point of the tube into the pulp-canal, compressing the bulb at the same time, and then allowing the bulb to expand to draw the air out again. In that way I get my canals very dry, and if I think there is likely to be trouble in getting the creamy oxychloride of zinc up to the apex, I take a piece of orange- or cedar-wood, a little smaller than the nerve-canal, and, after working in as much of the oxychloride as I can with the Donaldson nerve-broach, take the splinter of wood and drive it as far up as possible, and there leave it. I never make provision for opening a canal after having filled it; experience teaches me that it is unnecessary. If the root should afterwards give trouble, which very rarely happens, I know at once that I have not been thorough in the preliminary details.

I have opened a good many canals that have been filled with gutta-percha, and in a great many instances there has been considerable odor, and in some cases the gutta-percha itself has been in a putrescent condition. But in opening canals filled with the oxychloride of zinc, I have noticed an absence of odor, and believe that this substance renders inert any portion of decay that may have eluded my efforts to remove it. Of course, there are many methods of filling canals where the tooth has been devitalized and the pulp immediately removed, but I am speaking particularly of *putrescent* canals and my experience with oxychloride of zinc in such cases.

*President Brackett.*—The chair would like to clearly understand whether the last speaker feels justified in introducing this root-filling before the odor from the canal has disappeared, and before there is evidence of a general state of health of the investment of the root.

*Dr. Allen.*—I never introduce any root-filling until I have made myself reasonably sure that all products of decomposition have been removed. My method is to be as thorough in the first cleansing as possible, using peroxide of hydrogen to a large extent, and hook broaches and other canal cleaners to get out all the loose decayed substance. Then I leave a dressing within the root for from three to five days, saturated sometimes with carbolic acid, sometimes with nothing but peroxide of hydrogen, occasionally listerine, according to the amount of putrescence present. I find that I have occasion to depend less and less on these substances according as I am thorough in the removal of the decay.

I continue this treatment as long as the odor persists, and when there is an absence of odor, I proceed to fill the canal.

*Dr. Andrews.*—My method of treating these canals I suppose is the same as used by many others. I do not believe in over-treatment. My aim is to cleanse the canal as thoroughly as possible. I seldom treat more than three times.

I find a very excellent antiseptic to use is a mixture of oil of cassia, oil of wintergreen, and carbolic acid, combined according to the formula of Dr. Black. I don't remember the proportions. In filling the roots I use chloro-percha, or liquid gutta-percha, and gutta-percha in the form of points, usually oxyphosphate over that, as I seldom fill the tooth permanently at this time. The filling is allowed to remain for six months or a year before permanently filling the tooth.

Another matter I wish to speak about is this: Dr. Stanton, president of the Harvard Odontological Society, spoke to me of his success in using oil of cassia combined with iodoform, and stated that he had had several difficult cases of abscessed teeth which he had succeeded in curing by this treatment.

Some time after this, speaking to a friend who is a druggist, I asked him if oil of cassia and iodoform could not be combined with some medium to make it dense enough to be rolled into points, like gutta-percha points. He thought it could be done, and, after experimenting, he made some for me, which I have used in several troublesome cases with good success. How long it will be before they dissolve, or, if they ever do, I cannot tell.

The gutta-percha points, when carefully manipulated, are so well adapted for pulp-canal fillings that I should be loath to give them up.

*Dr. Eddy.*—I should like to ask Dr. Andrews whether he attributes the good properties of that mixture to the oil of cassia or to the iodoform?

*Dr. Andrews.*—To the oil of cassia.

*Dr. Eddy.*—Then why not use some antiseptic like hydronaphthol, and be free from the objectionable odor of iodoform?

*Dr. Stevens.*—I use tannic acid and creosote in root-canals, and seldom have failures.

*Dr. Eddy.*—I don't see why the dentist should tolerate in his office the odors from the old-fashioned antiseptics, and neither do I see why it is necessary that he should require a number of sittings to treat and fill a root-canal.

A lady came to me the other day with a second left inferior bicuspid that was very much inflamed. The crown and distal surfaces were filled with gold. There was an abscess pointing to the lingual side. I lanced the abscess, removed the dead pulp, and cleansed the canal, forcing peroxide of hydrogen through the fistulous opening. Redrying the canal, I forced a solution of chloropercha and oil of cassia through the tract. Inflammation soon subsided, and the patient has had no trouble.

*Dr. Stevens.*—I suppose the gentlemen referred to me when he spoke about the odor from old-fashioned antiseptics. If you put tannic acid and creosote together, you get no odor from them to permeate the office.

*Dr. Eddy.*—I did not refer to any one in particular, but you cannot cover those odors so they will not be noticed. We may not observe it ourselves, but there is an accumulation of odors that the patients notice.

*Dr. Williams.*—I remember Dr. Charles S. Tomes, some years ago, mentioned that, in opening a tooth in which the pulp had been dead for some time, he always kept it bathed in eucalyptus oil, which at one time had quite a reputation as an antiseptic. If we needed a stronger antiseptic, we might combine the oil of cassia with the eucalyptus oil. There are so many nice things in the list of antiseptics now, that it would seem to be very easy to make a selection.

*Dr. Andrews.*—I used iodoform experimentally. It was recommended highly, and I wanted to see if it worked right in my cases.

I remember reading about a dentist who used a mixture of one-half arsenic and one-half oxyphosphate of zinc, and filled the root-canals with it, and stated he could cure any case no matter how bad.

*Dr. Allen.*—I think the discussion has brought out some points that I should like to try, such, for instance, as the essential oils, with which I have but little experience.

*Dr. Andrews.*—I sometimes find that, after drying the canals, there is apt to be still a little moisture at the apex. I find traces of it on the cotton used.

*Dr. Allen.*—I think the solution of oxychloride of zinc overcomes that, as it acts as a cauterant.

William H. Potter, D.M.D.,
*Editor American Academy of Dental Science.*

